# Unintentional Mitral Lateral Isthmus Block During Pulmonary Vein Isolation With a Pentaspline Pulsed‐Field Ablation Catheter

**DOI:** 10.1002/joa3.70226

**Published:** 2025-11-17

**Authors:** Yuhei Kasai, Takayuki Kitai, Junji Morita, Kei Murakami, Kazuhiro Satomi

**Affiliations:** ^1^ Department of Cardiology, Sapporo Heart Center Sapporo Cardiovascular Clinic Sapporo Japan; ^2^ Department of Cardiology Tokyo Medical University Tokyo Japan

**Keywords:** atrial fibrillation, mitral isthmus block line, pulmonary vein isolation, pulsed‐field ablation

## Abstract

This case demonstrates an unintentional bidirectional mitral isthmus block created during pulmonary vein isolation using a pentaspline pulsed‐field ablation catheter. The finding underscores the importance of careful sheath–catheter alignment at the left inferior pulmonary vein to prevent unintended lesion extension toward the mitral annulus.

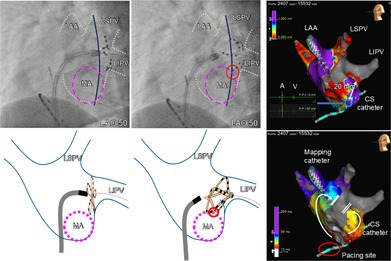

Pulsed‐field ablation (PFA) is a novel nonthermal technique for atrial fibrillation (AF) ablation that selectively targets myocardial cells while sparing surrounding noncardiac tissue. Recent clinical studies and real‐world data have demonstrated the procedural efficacy and reproducibility of pulmonary vein isolation (PVI) using the pentaspline PFA catheter (FARAWAVE, Boston Scientific, Marlborough, MA, USA) [1, 2]. In Japan, the clinical use of PFA is currently limited to PVI; however, in other countries, previous studies have reported the creation of mitral isthmus block lines using the FARAPULSE system [2, 3]. This typically required additional applications delivered in a flower configuration at the mitral isthmus. Here, we describe a unique case in which bidirectional mitral isthmus block was unintentionally achieved with a minimal number of applications.

A 60‐year‐old man with symptomatic paroxysmal AF underwent PFA using the FARAPULSE system (Boston Scientific) under general anesthesia (Figure 1). Following transseptal puncture using a 16.8‐Fr PFA sheath (Faradrive, Boston Scientific) and a radiofrequency guidewire (Versacross, Boston Scientific), left atrial angiography was performed with a pigtail catheter during right ventricular burst pacing (Videos S1 and S2). PVI was performed under fluoroscopic guidance (without 3D mapping) with the minimum number of applications: eight per pulmonary vein (four in the flower and four in the basket configuration, for a total of 32 applications). Post‐ablation mapping using the Ensite X system (Abbott, Chicago, IL, USA) during pacing from the coronary sinus (CS) ostium confirmed successful PVI and, unexpectedly, revealed a complete mitral isthmus block line (Figure 2A). This high‐density map, constructed using an HD Grid catheter (Abbott) with more than 2000 acquisition points, was considered sufficiently reliable to demonstrate that a true conduction block had indeed occurred across the mitral isthmus. Subsequent pacing from a mapping catheter positioned in the left atrial appendage showed a proximal‐to‐distal activation sequence in the CS (Figure 2B). These findings confirmed bidirectional conduction block across the mitral isthmus. No major ST‐segment changes were observed during the procedure, which was completed without any complications. During the 6‐month follow‐up, the patient remained free of AF and atrial tachycardia.

**FIGURE 1:**
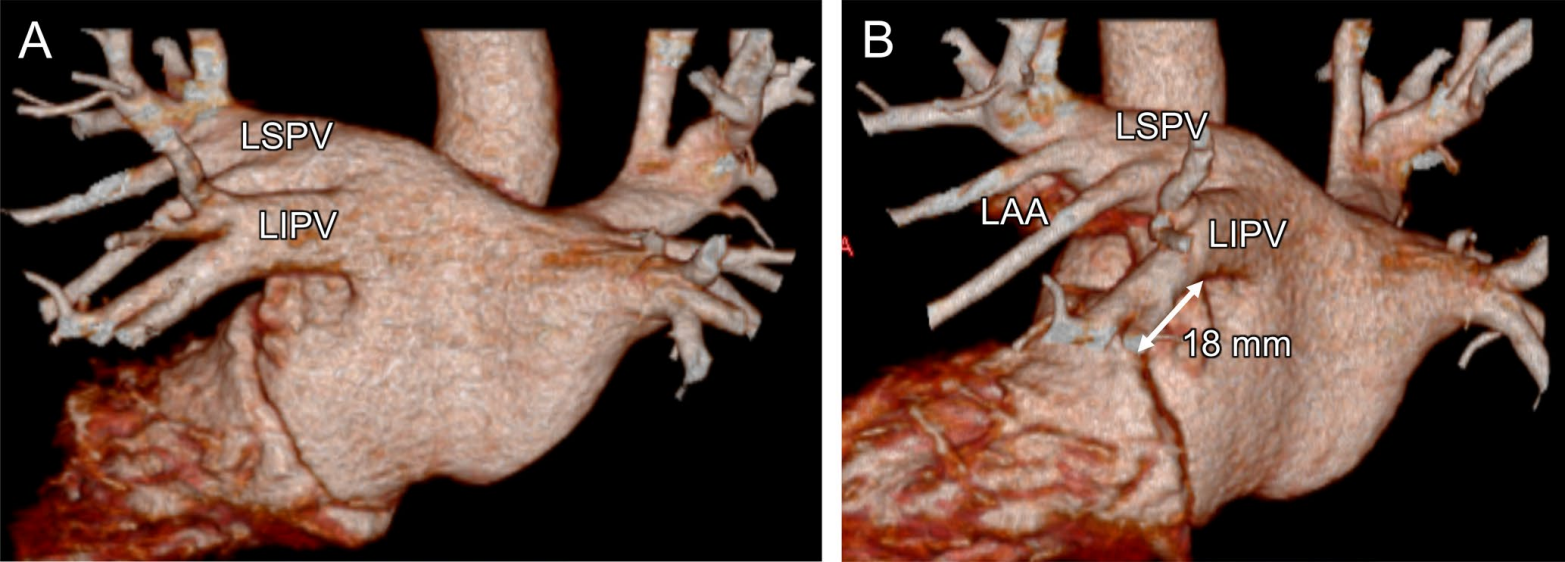
Three‐dimensional computed tomography of the pulmonary veins before the procedure. (A) Posterior–anterior view. (B) Left posterior oblique view. The white arrow indicates the mitral isthmus, which measures approximately 18 mm in length. LSPV, left superior pulmonary vein; LIPV, left inferior pulmonary vein; LAA, left atrial appendage.

**FIGURE 2:**
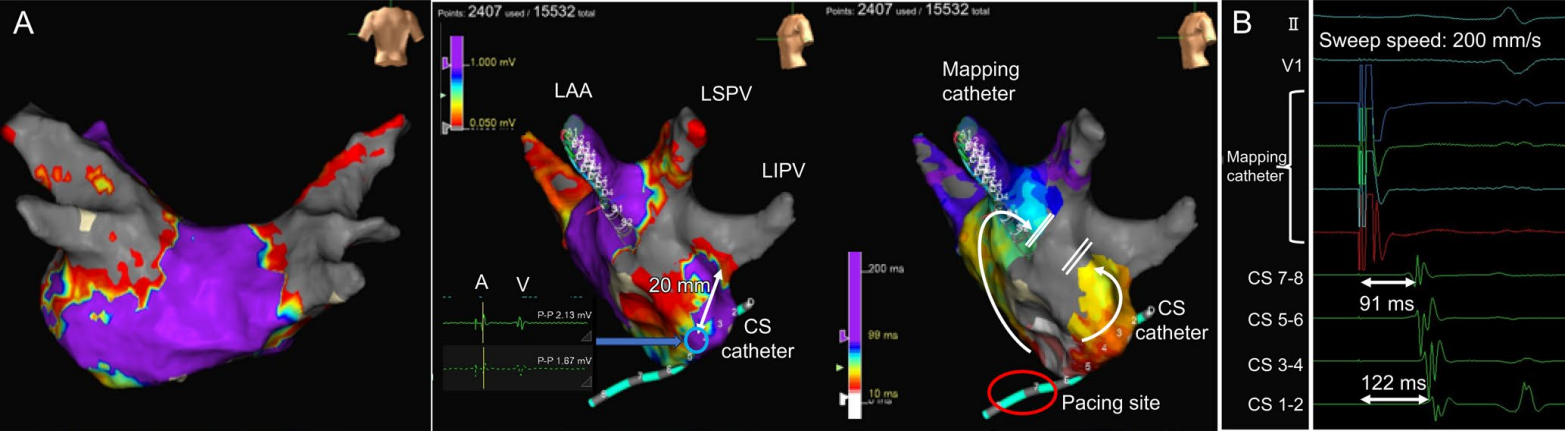
Post‐ablation mapping and intracardiac echocardiogram. (A) Post‐ablation mapping during CS ostium pacing shows a complete mitral isthmus block line. Left panel: voltage map (posterior–anterior view); middle panel: voltage map (left anterior oblique view); right panel: activation map (left anterior oblique view). (B) Pacing from the mapping catheter located in the LAA shows a proximal‐to‐distal activation pattern in the CS. LSPV, left superior pulmonary vein; LIPV, left inferior pulmonary vein; LAA, left atrial appendage; CS, coronary sinus.

Although previous studies have reported the creation of a mitral isthmus block line using the FARAPULSE system, this typically required additional applications in a flower configuration at the mitral isthmus [2, 3]. To the best of our knowledge, this is the first reported case in which a bidirectional mitral isthmus block was achieved with a minimum number of applications (32 applications). A retrospective review of fluoroscopic images (Figure 3) and schematic illustration (Figure 4) showed that (1) the left superior pulmonary vein applications did not appear to have contributed to the creation of the mitral isthmus block and (2) the PFA applications in the basket configuration at the left inferior pulmonary vein (LIPV) were delivered slightly more anteriorly and proximally than those in the flower configuration.

**FIGURE 3:**
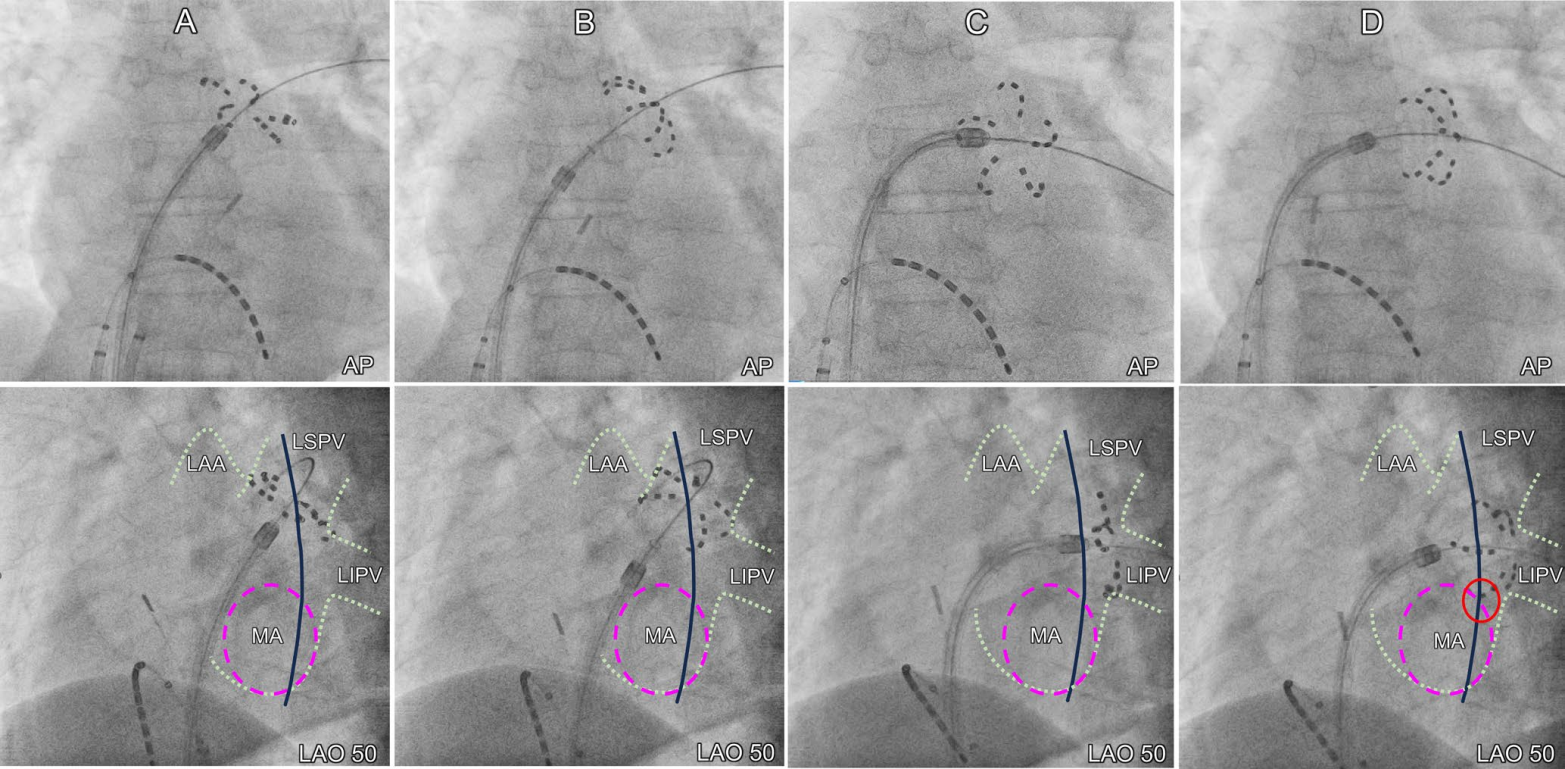
Fluoroscopic comparison of flower and basket configurations at the left pulmonary veins. Fluoroscopic images of (A) the LSPV in the flower configuration, (B) the LSPV in the basket configuration, (C) the LIPV in the flower configuration, and (D) the LIPV in the basket configuration. The upper panels show the AP view, and the lower panels show the LAO view. The blue curved line marks the right border of the vertebral body, whereas the green and red dashed lines delineate the LA–PV junction and the MA, respectively. In the LIPV, the basket configuration is located slightly more anteriorly than the flower configuration. The red circle in panel (D) indicates the region where the application lesion extended to the MA. LSPV, left superior pulmonary vein; LIPV, left inferior pulmonary vein; LAA, left atrial appendage; MA, mitral anulus; AP, anteroposterior; LAO, left anterior oblique; LA, left atrium; PV, pulmonary vein.

**FIGURE 4:**
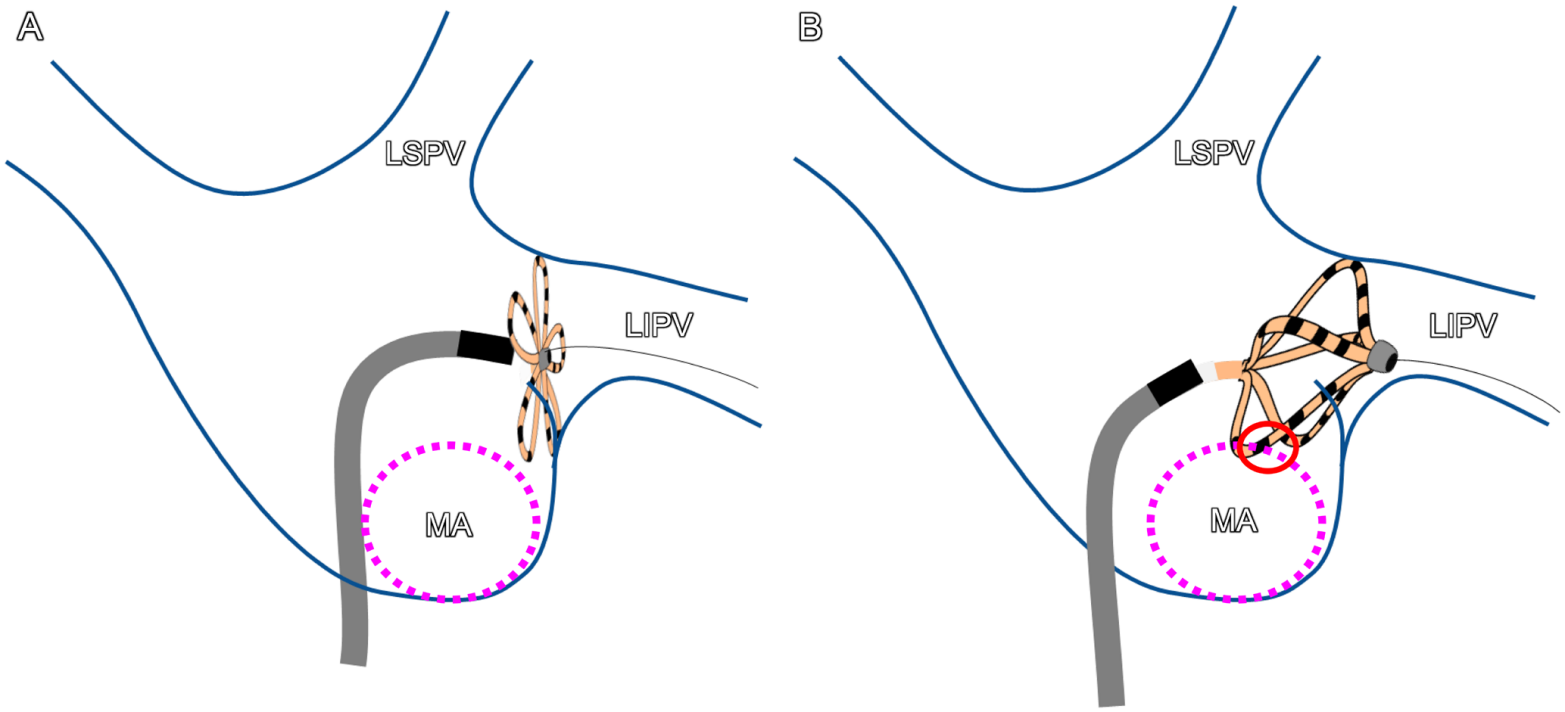
Schematic illustration corresponding to the fluoroscopic images of the LIPV shown in Figure 3C and D. (A) Corresponds to Figure 3C, showing the flower configuration at the LIPV. (B) Corresponds to Figure 3D, showing the basket configuration. In the LIPV, the basket configuration is positioned slightly anterior to the flower configuration. The red circle in panel (B) highlights the area where the ablation lesion reached the MA. LSPV, left superior pulmonary vein; LIPV, left inferior pulmonary vein; MA, mitral anulus.

The cause of the unintentional lesion formation is considered to be related to suboptimal alignment between the FARADRIVE sheath and the FARAWAVE catheter during the LIPV applications. In Figure 3, the tip of the FARADRIVE sheath is clearly oriented toward the anterior wall, suggesting that the catheter was inadvertently pulled anteriorly, thereby extending the application toward the lateral mitral isthmus. This misalignment would have been easier to recognize in the right anterior oblique view, whereas in the AP view it can remain subtle when using the basket configuration. Particularly during applications at the LIPV, it would have been preferable to reposition the CS catheter into the CS to serve as an anatomical landmark. Additionally, the naturally short length (18 mm in computed tomography, Figure 1B; 20 mm in 3D mapping, Figure 2A) of the mitral isthmus may have facilitated this unexpected outcome. The durability of the lesion formation in the LIPV and the unintentional mitral isthmus block line remains uncertain, as neither AF nor atrial tachycardia has recurred, and no repeat ablation procedure has been undertaken.

This case highlights the importance of careful attention to catheter–sheath alignment and anatomical orientation during PFA applications at the LIPV to prevent unintentional mitral isthmus lesion formation—a phenomenon that cannot occur with PVI performed using radiofrequency ablation.

## Ethics Statement

This research was conducted according to the principles of the Declaration of Helsinki.

## Consent

The patient provided written informed consent for publication of the details of her case.

## Conflicts of Interest

The authors declare no conflicts of interest.

## Supporting information


**Video S1:** Left atrial angiography performed with a pigtail catheter during right ventricular burst pacing (anteroposterior view).

**Video S2:** Left atrial angiography performed with a pigtail catheter during right ventricular burst pacing (left anterior oblique view).

## Data Availability

The authors confirm that all the data supporting the findings of this research is available within the article.
